# RUNX3-mediated circDYRK1A inhibits glutamine metabolism in gastric cancer by up-regulating microRNA-889-3p-dependent FBXO4

**DOI:** 10.1186/s12967-022-03286-x

**Published:** 2022-03-10

**Authors:** Haofeng Liu, Qiu Xue, Hongzhou Cai, Xiaohui Jiang, Guangxin Cao, Tie Chen, Yuan Chen, Ding Wang

**Affiliations:** 1 Department of General Surgery, Tumor Hospital Affiliated to Nantong University & Nantong Tumor Hospital, No. 30, Tongyang North Road, Pingchao Town, Tongzhou District, Nantong, 226361 Jiangsu People’s Republic of China; 2 Department of Urology, Jiangsu Cancer Hospital & Jiangsu Institute of Cancer Research &, The Affiliated Cancer Hospital of Nanjing Medical University, Nanjing, 210009 People’s Republic of China

**Keywords:** RUNX3, CircDYRK1A, Gastric cancer, Glutamine metabolism, MicroRNA-889-3p, FBXO4, Sponge

## Abstract

**Background:**

Targeting glutamine metabolism is previously indicated as a potential and attractive strategy for gastric cancer (GC) therapy. However, the underlying mechanisms responsible for the modification of glutamine metabolism in GC cells have not been fully elucidated. Accordingly, the current study sought to investigate the physiological mechanisms of RUNX3-mediated circDYRK1A in glutamine metabolism of GC.

**Methods:**

Firstly, GC tissues and adjacent normal tissues were obtained from 50 GC patients to determine circDYRK1A expression in GC tissues. Next, the binding affinity among RUNX3, circDYRK1A, miR-889-3p, and FBXO4 was detected to clarify the mechanistic basis. Moreover, GC cells were subjected to ectopic expression and knockdown manipulations of circDYRK1A, miR-889-3p, and/or FBXO4 to assay GC cell malignant phenotypes, levels of glutamine, glutamic acid, and α-KG in cell supernatant and glutamine metabolism-related proteins (GLS and GDH). Finally, nude mice were xenografted with GC cells to explore the in vivo effects of circDYRK1A on the tumorigenicity and apoptosis.

**Results:**

circDYRK1A was found to be poorly expressed in GC tissues. RUNX3 was validated to bind to the circDYRK1A promoter, and circDYRK1A functioned as a miR-889-3p sponge to up-regulate FBXO4 expression. Moreover, RUNX3-upregulated circDYRK1A reduced levels of glutamine, glutamic acid, and α-KG, and protein levels of GLS and GDH, and further diminished malignant phenotypes in vitro. Furthermore, in vivo experimentation substantiated that circDYRK1A inhibited the tumorigenicity and augmented the apoptosis in GC.

**Conclusion:**

In conclusion, these findings highlighted the significance and mechanism of RUNX3-mediated circDYRK1A in suppressing glutamine metabolism in GC via the miR-889-3p/FBXO4 axis.

**Supplementary Information:**

The online version contains supplementary material available at 10.1186/s12967-022-03286-x.

## Background

Gastric cancer (GC) remains the third leading cause of cancer-associated deaths across the world [1]. Surgery is regarded the gold-standard for GC treatment, and multimodality treatment approach is needed to further augment the survival rate [2]. Interestingly, new findings have to come to highlight the potential of glutamine inhibition to target cancer cells due to the critical biosynthetic and energy-generating roles of glutamine in cancer cell growth [3, 4]. In addition, humanized monoclonal antibody targeting glutamine transporter ASCT2 was previously shown to suppress GC patient-derived xenografts via blocking the cellular glutamine metabolism [5]. Accordingly, the discovery of novel therapeutic targets associated with glutamine metabolism in GC may pave the way for the development of more effective therapies for GC.

RUNX3 belongs to the runt domain family of transcription factors [6], and a number of researchers have explored its roles in GC progression [7]. The TRCirc database further suggests that RUNX3 binds to the promoter region of circDYRK1A at (chr21:38,780,489–38,780,655). Meanwhile, circRNAs represent a member of the noncoding RNA family [8], which may function as oncogenes or tumor suppressor genes in the progression of GC, which provides a promising therapeutic target or biological drug for the treatment of GC [9]. Furthermore, existing evidence indicates that circRNAs can also participate in GC development due to their ability to regulate GC cell proliferation and invasion [10]. Moreover, a recent study has validated the participation of circRNAs in the development of cancer by virtue of regulating glutamine metabolism [11].

Furthermore, initial bioinformatics analyses in our study suggested that circDYRK1A could be capable of sponging microRNA-889-3p (miR-889-3p). CircRNAs are known to function as ceRNAs, which is critical for the circuitry of microRNAs (miRNAs) and their target genes [12]. Notably, these circRNAs, acting as a ceRNA to regulate miRNAs, play a significant role in GC carcinogenesis [13]. On the other hand, miRNAs, well-established as small, non-coding RNAs, can exert effects by inhibiting the target messenger RNA translation and confer crucial roles in the pathogenesis of numerous human diseases by regulating their specific target activity [14]. Moreover, dysregulation of miRNAs has been previously shown to serve as oncogenes or tumor suppressors in regard to GC [15]. However, the specific mechanisms of circDYRK1A sponging miR-889-3p in GC and their function in regulating glutamine metabolism remain elusive. Accordingly, we speculated a potential mechanism by which the RUNX3-mediated circDYRK1A may bind to the miR-889-3p, thereby influencing GC development.

## Methods

### Bioinformatics analysis

The GC-related microarray datasets GSE93541 (circRNA), GSE89143 (circRNA), GSE49051 (mRNA) and GSE54397 (miRNA) were obtained from the GEO database. The R "limma" package was adopted for differential analysis, with ∣logFC∣ > 2, *p* < 0.05 serving as the threshold to screen the significant differential expression of circRNA. The TRCirc database was employed to predict the transcription factors that regulate circRNAs. The Circumtactome database was utilized to predict miRNAs that circRNA binds to. The starBase database was employed to predict target genes of miRNA.

### Study subjects

GC and adjacent normal tissue specimens were obtained from a total of 50 GC patients, confirmed with primary GC by endoscopy and underwent radical operation at the Tumor Hospital Affiliated to Nantong University & Nantong Tumor Hospital from 2015 to 2017. None of the included patients previously underwent surgery, radiotherapy, or chemotherapy prior to specimen collection. All the obtained specimens were promptly frozen in liquid nitrogen and stored at − 80 °C. The significance and prognostic value of circDYRK1A were both analyzed in the abovementioned 50 pairs of specimens with clinicopathological parameters. Clinical characteristics of the included patients are presented in Additional file 1: Table S1. The current study was approved by the Ethics Committee of Tumor Hospital Affiliated to Nantong University & Nantong Tumor Hospital and carried out in strict accordance with the *Declaration of Helsinki*. All participants signed informed consent documentation before sample collection.

### In situ hybridization (ISH)

The collected tissue sections were dewaxed, rehydrated, fixed with 4% paraformaldehyde and digested with proteinase K. Subsequently, the sections were hybridized with digoxin-labeled circDYRK1A probe overnight, followed by overnight incubation with digoxin resistant mAb (Roche, Switzerland) at 4 °C. The following day, the sections were stained with NBT/BCIP.

### Immunohistochemistry

The aforementioned paraffin samples were sectioned and immersed in 3% methanol H_2_O_2_. Next, the sections were retrieved in antigen retrieval solution, followed by sealing in normal goat serum blocking solution at room temperature for 20 min. Subsequently, the sections were probed with the primary anti-rabbit polyclonal anti-GLS antibody (ab260047, dilution ratio of 1: 200, Abcam, Cambridge, UK), GDH (ab170895, dilution ratio of 1: 100, Abcam), RUNX3 (ab224642, dilution ratio of 1: 1000, Abcam) and FBXO4 (ab230302, dilution ratio of 1:1000, Abcam) at 4 °C overnight. The following day, the sections were incubated with the secondary antibody IgG (goat anti-rabbit, ab6721, dilution ratio of 1: 1000, Abcam) at 37 °C for 20 min. Afterwards, the sections were stained with DAB (ST033, Weijia Technology) development, and then counter-stained with hematoxylin. Thereafter, the sections were visualized under a microscope (CX43, Olympus Optical Co., Ltd., Tokyo, Japan). The Nikon image analysis software was utilized to document the positive cells. A total of 5 non-repetitive visual fields of equal area (200 times) were selected from each section and the number of positive cells and their proportions were calculated, with the average value calculated.

### Cell culture and transfection

GC cell lines (namely, AGS, MKN74, HGC-27, N87, SNU-5, and HS-746 T) and normal human gastric epithelial cell lines (namely, GES-1 and 293 T), all purchased from Shanghai Institute of Biological Sciences, (Shanghai, China) were cultured in DMEM (10% FBS, 100 U/mL penicillin and 100 U g/mL streptomycin).

GC cells (2–6 × 10^5^ cells/well) were plated in 6-well plates, and then transfected using the Lipofectamine 3000 reagent (Invitrogen, Carlsbad, CA) or an Adeno-X Adenoviral System 3 (Clontech, Mountain View, CA). The cells were transduced with vector, circDYRK1A, oe-NC, oe-RUNX3, sh-NC, sh-FBXO4-1, sh-FBXO4-2, sh-NC + vector, sh-FBXO4 + vector, sh-FBXO4 + circDYRK1A, inhibitor-NC + vector, miR-889-3p inhibitor + vector, miR-889-3p inhibitor + circDYRK1A, sh-circDYRK1A-1, sh-circDYRK1A-2, sh-RUNX3-1, sh-RUNX3-2, mimic-NC + sh-NC, miR-889-3p mimic + sh-NC, or miR-889-3p mimic + sh-circDYRK1A. The expression plasmid (50 ng/mL) was purchased from GenePharma Co., Ltd. (Shanghai, China).

Construction of the lentiviral packaging system was carried out with LV5-GFP and pSIH1-H1-copGFP. The packaging virus related plasmids were co-transfected into 293 T cells. The supernatant was collected after 48 h of cell culture, and the supernatant was filtered and centrifuged to contain virus particles. The related groups were as follows: HGC-27 cells: lv-oe-NC + sh-NC, lv-oe-RUNX3 + sh-NC, lv-oe-NC + sh-circDYRK1A, lv-oe-RUNX3 + sh-circDYRK1A. The cells at the logarithmic growth phase were digested with trypsin, and then prepared into a cell suspension containing 5 × 10^4^ cells/mL. Subsequently, the cell suspension was seeded in a 6-well plate, 2 mL per well, and cultured overnight at 37 °C. The plasmids (lv-oe-NC, lv-oe-RUNX3, sh-NC and sh-circDYRK1A) were purchased from Shanghai GenePharma Co., Ltd. (Shanghai, China) and the concentration of all plasmids was 50 ng/mL.

### RT-qPCR

The TRIzol reagent (Article No. 16096020, Thermo Fisher technology, New York) was adopted for total RNA extraction. For detection of mRNA, reverse transcription kits (RR047A, Takara, Japan) were utilized to obtain cDNA. For miRNA, PCR kits (B532451, Sangon Biotechnology) containing Universal PCR primer F and U6 Universal PCR primer R were employed to obtain the cDNA of miRNA containing PolyA tail. SYBR® Premix Ex TaqTM II kits (DRR081, Takara, Japan) combined with qPCR instrument (ABI 7500; Applied Biosystems, Foster City, CA) was utilized for RT-qPCR detection. U6 mRNA levels were regarded used as internal reference.

RT-qPCR was performed as per TaqMan Gene Expression Assays protocols (Applied Biosystems). Three replicates were set for RT-qPCR. With GAPDH serving as internal reference, the 2^−ΔΔCt^ method was adopted to quantify expression of target genes. The primer sequences are shown in Additional file 3: Table S2.

### Western blot analysis

RIPA lysis buffer (Beyotime) was adopted for total protein content extraction, with protein concentration assessed using BCA kits. Next, the proteins were separated by SDS-PAGE, and then transferred onto PVDF membrane (IPVH85R, Millipore, Darmstadt, Germany). After blocking, the membranes were incubated with specific primary antibodies, rabbit anti-human GLS (ab260047, dilution ratio of 1: 250, Abcam), GDH (ab170895, dilution ratio of 1: 100, Abcam), FBXO4 (ab230302, dilution ratio of 1:1000, Abcam), and GAPDH (ab181602, dilution ratio of 1:10,000, Abcam) at 4 °C overnight. The following day, the membranes were incubated with HRP-labeled IgG (goat anti-rabbit, ab205718, dilution ratio of 1: 5000, Abcam) for 1 h at room temperature. ECL was adopted to visualize the results, with band intensities quantified using the ImageJ software (1.48 u, National Institutes of Health, Bethesda, Maryland). Relative protein expression = gray values of the target protein band / gray values of internal reference GAPDH band.

### EdU assay

The cells were seeded in 24-well plates, and incubated for 2 h with EdU (10 µmol/L). Next, the cells were treated with 100 µL dye solution in conditions void of light for 30 min at room temperature. DAPI was adopted for nuclear staining for 5 min, after which the cells were observed under a fluorescence microscope (FM-600, Putan Optical Instrument Co., Ltd., Shanghai, China) in 6–10 randomly chosen fields. Afterwards, positive cells were counted in each field.

### Transwell assay

A Transwell chamber (8 mm aperture; Corning, NY) was adopted for detection of cell migration and invasion in a 24-well plate. In the Transwell chamber without Matrigel or with Matrigel, 600 ml of 20% FBS culture medium was added in advance and equilibrated at 37 °C for 1 h. Subsequently, AGS and HGC-27 cells were resuspended in the FBS-free medium, followed by being plated in the upper chamber (1 × 10^6^ cells/mL) and cultured. Next, the chamber was fixed with 5% glutaraldehyde, and stained with 0.1% crystal violet for 5 min at 4 °C. The surface cells were wiped off with cotton ball. Afterwards, the cells were observed under a Nikon inverted fluorescence microscope (TE2000). The number of positive cells in each field was recorded and statistically analyzed, with average value acquired.

### Luciferase reporter assay

FBXO4 3′UTR and miR-889-3p binding site mutant particles: pmirGLO-FBXO4-WT and pmirGLO-FBXO4-MUT were constructed respectively. Next, the reporter plasmids were co-transfected with miR-889-3p mimic and NC plasmid into 293 T cells, respectively. After 48 h, the cells were lysed, and collected. Dual-Luciferase® Reporter Assay System was subsequently used to examine luciferase activity, which was represented as: Firefly luciferase activity/Renilla luciferase activity. 100 μL firefly luciferase working solution (RG005, Beyotime Biotechnology Co., Shanghai, China) was added to each cell sample.

The TRCirc database was utilized to predict the binding site of RUNX3 protein to circDYRK1A DNA. The recombinant luciferase reporter gene vector with mutation binding site was constructed, and then co-transfected with RUNX3 expression vector into 293 T cells. The specific binding site of RUNX3 protein to circDYRK1A DNA was verified with a double luciferase reporter experiment. The specific method and steps were the same as above.

#### FISH

Localization of circDYRK1A in cells was examined as follows: biotin-labeled specific RNA probes were transcribed from circDYRK1A PCR fragments. Following growth to the exponential phase, the cells were fixed with 4% formalin. Next, the cells were hybridized with circDYRK1A specific biotin-labeled probe in hybridization buffer. The signal was measured with tyramine conjugated Alexa 488 fluorescence TSA kits. Photographs were taken with a laser scanning confocal microscope.

#### Measurement of levels of glutamine, glutamic acid and α-KG

Glutamine and glutamic acid concentrations were determined using glutamine/glutamic acid assay kits (GLN1-1KT, Sigma). The content of α-KG was determined using detection kits (ab83431, Abcam).

#### ChIP assay

EZ-Magna ChIP TMA kits (Millipore company, USA) were adopted for ChIP assay. The aforementioned cells were fixed with formaldehyde to cross-link the target protein. Glycine was then added to the cells and left to stand at room temperature for 5 min to terminate the fixation. Subsequently, the cells were centrifuged, lysed and randomly sonicated into 500–1000 bp fragments. Next, the cells were incubated overnight with the following antibodies at 4 °C: positive control RNA polymerase II, the NC antibody normal rabbit IgG and rabbit anti-RUNX3 (dilution ratio of 1: 25, ab224641, Abcam). The following day, protein Agarose/Sepharose was carried out to precipitate endogenous DNA–protein complexes, which was thereafter incubated with 5 M NaCl at 65 °C overnight for de-cross-linking. CircDYRK1A promoter expression was detected via qPCR, and the primer sequences are as follows: forward: 5′-TGTGGCTGACAGGTAATGTGG-3′ and reverse: 5′-GGACACATGGTAAGTGTGCAA-3′.

#### Rnaser processing

AGS and HGC-27 cells were treated with RNase R (4 U/mg, Epicenter) at 37 °C for 30 min, whereupon the treated RNA was reverse-transcribed with specific primers and examined using qPCR.

#### RIP assay

RIP assay was carried out with magna RIP TM RIP kits (Millipore). Briefly, AGS and HGC-27 cells were lysed and then incubated with RIP buffer containing NC IgG or AGO2 antibody (mouse, Millipore) coupled magnetic beads overnight. The following day, after incubation with proteinase K for 30 min, the immunoprecipitation RNA content was extracted. Later, the expression of circDYRK1A and miR-889-3p was detected using qPCR.

#### RNA pull-down assay

In order to determine the circRNA pulled-down by miRNA, AGS and HGC-27 cells were lysed and treated with over-expressing circDYRK1A, followed by incubation with miR-889-3p biotin coupled probes pre-attached to magnetic beads. Thereafter, RNA content was extracted using RNeasy MiniKits (QIAGEN, Germany) for 2 h. Afterwards, the pull-down products were extracted and the expression of circDYRK1A was detected by qPCR.

#### In vivo tumor formation assay

A total of 24 male BALB/c mice (aged 6 weeks old) were included for in vivo studies from the Institute of Zoology, Chinese Academy of Sciences. The nude mice were subcutaneously injected in the armpit with HGC-27 cells (2 × 10^6^) transduced with lv-oe-NC + sh-NC, lv-oe-RUNX3 + sh-NC, lv-oe-NC + sh-circDYRK1A, and lv-oe-RUNX3 + sh-circDYRK1A. From the 7th day, the tumor volume was measured with digital calipers every 3 days, and calculated as follows: length × width^2^ × 0.5. After 28 days, all the injected nude mice were euthanized, whereupon the weight of tumor was measured, and the tumor tissues were visualized by TUNEL and immunohistochemical staining. Animal experimentation was approved by the Ethics Review Committee for Tumor Hospital Affiliated to Nantong University & Nantong Tumor Hospital.

#### TUNEL staining

The xenografted mouse tumor tissues were sectioned, after which the sections were stained with TUNEL, and heated at 60 °C for 15 min, followed by dewaxing and hydration. Following 10-min treatment with protease K solution at room temperature, the sections were then incubated for 1 h with the TUNEL reagent at 37 °C. Subsequently, the sections were treated with 3% H_2_O_2_ methanol solution at ambient temperature. After incubation with invert peroxidase solution at 37 °C for 30 min, the sections were stained with DAB, and rinsed with PBS. Afterwards, the sections were stained with hematoxylin, followed by dehydration with gradient ethanol, permeabilized with xylene, and sealed with neutral gum. The TUNEL positive cells were yellow brown under a light microscope.

### Statistical analysis

Statistical analyses were processed using the SPSS 21.0 software. Measurement data were described as mean ± standard deviation. Statistical significance was measured using paired *t*-test (paired data), unpaired *t*-test (unpaired data), one-way ANOVA (multi-group data) or repeated measures ANOVA (multi-group data) with Tukey’s multiple comparisons test. A value of *p* < 0.05 was regarded statistically significant.

## Results

### CircDYRK1A is down-regulated in GC, and further associated with the prognosis of GC patients

**Fig. 1:**
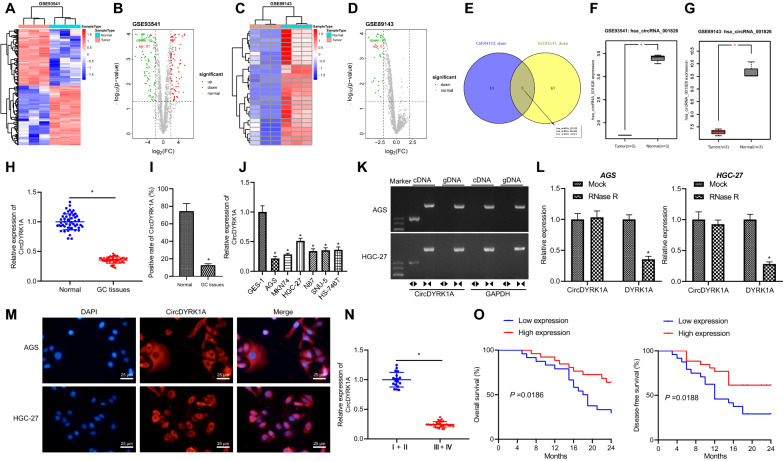
CircDYRK1A is poorly expressed in GC tissues and cells. **A** Unsupervised heat map of differentially expressed miRNAs in microarray dataset GSE93541. **B** Volcano map of differentially expressed miRNAs in microarray dataset GSE93541. **C** Unsupervised thermogram of differentially expressed miRNAs in microarray dataset GSE89143. **D** The volcano map of differentially expressed miRNAs in microarray dataset GSE89143. **E** Venn diagram of downregulated circRNAs in microarray datasets GSE93541 and GSE89143. **F** The expression of hsa_circRNA_001826 (circDYRK1A) in microarray dataset GSE9354. **G** The expression of hsa_circRNA_001826 (circDYRK1A) in microarray dataset GSE89143. **H** Expression of circDYRK1A in GC tissues and adjacent normal tissues (n = 50) determined RT-qPCR. **I** Expression of circDYRK1A in GC tissues and adjacent normal tissues detected by ISH assay. **J** Expression of circDYRK1A in normal gastric mucosa cell line GES-1 and GC cell lines AGS, MKN74, HGC-27, N87, SNU-5 and HS-746 T determined RT-qPCR. **K** The splicing form of circDYRK1A in AGS cells and HGC-27 cells detected by gel electrophoresis.  indicates the amplification results of divergent primers and  indicates the amplification results of convergent primers. **L** Expression of circDYRK1A and DYRK1A in AGS cells and HGC-27 cells in the presence or absence of RNase R measured by RT-qPCR. **M** The location of circDYRK1A in AGS and HGC-27 cells detected by FISH assay. **N** Expression of circDYRK1A in patients with early GC (I + II) (n = 18) and patients with advanced GC (III + IV) (n = 32) detected by RT-qPCR. **O** The overall survival (OS) and disease-free survival (DFS) time of patients with GC analyzed by Kaplan–Meier analysis. The cell experiment was repeated three times. * *p* < 0.05 vs. adjacent normal tissues, GES-1 cells or Mock, or stage I + II

In order to explore the differentially expressed circRNA in GC, the limma package was adopted for differential analysis of the GSE93541 (circRNA), GSE89143 (circRNA), GSE49051 (mRNA) and GSE54397 (miRNA) microarray datasets obtained from the GEO database. Unsupervised cluster analysis heat maps of differentially expressed circRNA in microarray datasets GSE93541 and GSE89143 were drawn respectively (Fig. 1A, C). It was found that there were a total of 166 differentially expressed circRNAs, comprising of 81 up-regulated miRNAs and 85 down-regulated circRNAs in the GSE93541 microarray dataset (Fig. 1B). Meanwhile, there were 26 differential circRNAs, including 0 up-regulated circRNAs and 26 down-regulated circRNAs in the GSE89143 microarray dataset (Fig. 1D). The subsequent intersection of two microarray down-regulated expression of circRNAs was regarded as the candidate research objects (Fig. 1E). Three circRNAs (namely, hsa_circRNA_101592, hsa_circRNA_001826 and hsa_circRNA_102417) were obtained, which was in accordance with previously published literature [16]. Among them, hsa_circRNA_001826 (also known as hsa_circ_0001190) exhibited the most significant difference between the two microarray datasets (Additional file 1: Table S3). Moreover, the hsa_circRNA_001826 was poorly expressed in the GSE93541 and GSE89143 datasets (Fig. 1F, G). According to analysis of the circBase database, hsa_circRNA_001826 was located at chr21: 38,792,600–38,794,168, and also known as circDYRK1A.

Moreover, the results of RT-qPCR displayed that the expression of circDYRK1A in GC tissues was lower than that in adjacent normal tissues (Fig. 1H). In addition, ISH illustrated that circDYRK1A expression was also decreased in GC tissues (Fig. 1I). In addition, RT-qPCR results demonstrated that relative to GES-1, circDYRK1A expression was lower in GC cell lines, with the lowest expression in AGS cell line and the highest in HGC-27 cell line (Fig. 1J). Accordingly, we selected AGS and HGC-27 cells for following experimentation.

In order to further detect whether the head tail splicing of circDYRK1A is trans splicing or genome rearrangement, cDNA and gDNA content were extracted from AGS and HGC-27 cells and then subjected to amplification with convergent primers and divergent primers, respectively. circDYRK1A expression was detected only in cDNA and not detected in gDNA (Fig. 1K), indicating that the ring structure of circDYRK1A originated from reverse splicing. As illustrated in Fig. 1L, circDYRK1A resisted the degradation of RNase R, whereas there was rapid degradation in the linear form of DYRK1A. Afterwards, we detected the localization of circDYRK1A in AGS and HGC-27 cells by FISH and found that circDYRK1A was primarily expressed in the cytoplasm (Fig. 1M).

Furthermore, there were changes in the expression of circDYRK1A with varying GC staging. The expression of circDYRK1A in patients with early GC (I + II) was higher than that in patients with advanced GC (III + IV) (Fig. 1N, Additional file 1: Table S1). In addition, the results of Kaplan–Meier analysis indicated that patients with higher expression of circDYRK1A presented with longer OS and DFS than the patients with lower expression of circDYRK1A (Fig. 1O). Together, the abovementioned findings suggested that circDYRK1A was amplified in GC tissues and may serve as a prognostic marker for GC.

### CircDYRK1A inhibits malignant phenotypes of GC cells

**Fig. 2:**
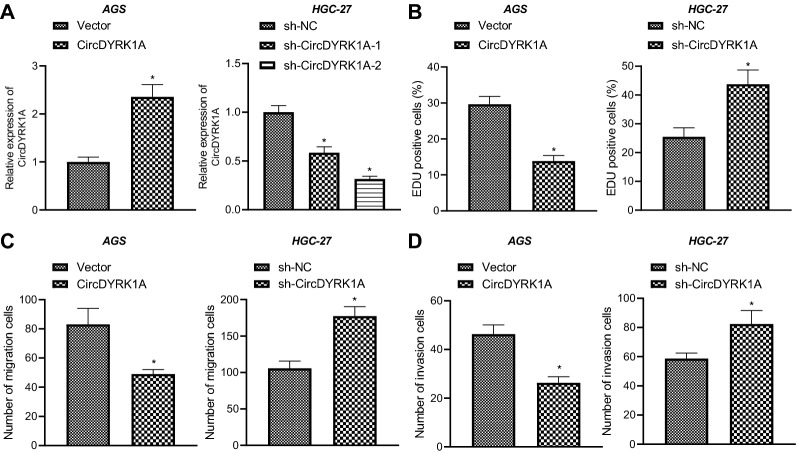
Overexpression of circDYRK1A inhibits malignant phenotypes of GC cells in vitro. AGS cells were transduced with circDYRK1A overexpression vector and HGC-27 cells were transduced with sh-circDYRK1A. **A** Expression of circDYRK1A in AGS and HGC-27 cells determined by RT-qPCR. **B** The proliferation of AGS and HGC-27 cells detected by EdU assay. **C** The migration of AGS and HGC-27 cells detected by Transwell assay. **D** The invasion of AGS and HGC-27 cells detected by Transwell assay. The cell experiment was repeated three times. * *p* < 0.05 vs. sh-NC-treated HGC-27 cells or Vector-treated AGS cells

We moved to further elucidate the effects of circDYRK1A on the malignant phenotypes of GC cells. The results showed that (Fig. 2A, Additional file 2: Fig. S1A) relative to HGC-27 and AGS cells transduced with vector, the expression of circDYRK1A was elevated in HGC-27 and AGS cells transduced with circDYRK1A over-expression vector. Meanwhile, circDYRK1A expression was decreased in HGC-27 and AGS cells transduced with sh-circDYRK1A-1 or sh-circDYRK1A-2, and the knockdown efficiency of sh-circDYRK1A-2 was higher. Therefore, sh-circDYRK1A-2 was selected for subsequent experimentation. To further validate the effects of circDYRK1A on the malignant phenotypes of GC cells, we carried out EdU and Transwell assays (Fig. 2B–D, Additional file 2: Fig. S1B–D) and uncovered that the HGC-27 and AGS cell proliferation, migration and invasion were inhibited by circDYRK1A over-expression, whereas the proliferative, migratory and invasive phenotypes of HGC-27 and AGS cells were all increased by silencing of circDYRK1A. Overall, these findings suggested that circDYRK1A could restrict the malignant phenotypes of GC cells.

### CircDYRK1A inhibits glutamine metabolism in GC cells

**Fig. 3:**
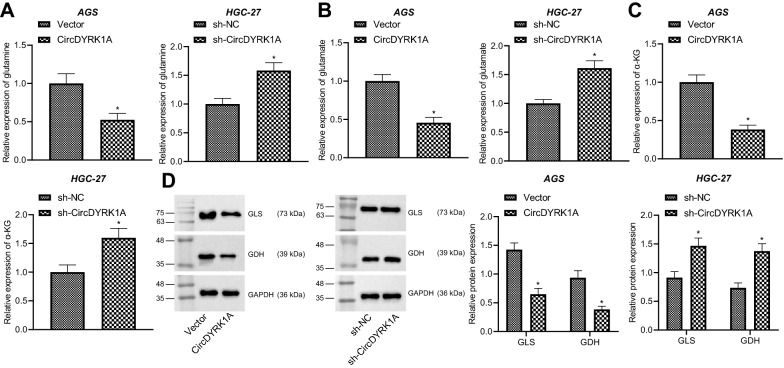
CircDYRK1A inhibits glutamine metabolism in GC cells. AGS cells were transduced with circDYRK1A overexpression vector and HGC-27 cells were transduced with sh-circDYRK1A. **A** The expression of glutamine in AGS and HGC-27 cells detected using the kit. **B** The expression of glutamic acid in AGS and HGC-27 cells detected using the kit. **C** The expression of α-KG in AGS and HGC-27 cells detected using the kit. **D** The protein levels of GLS and GDH in AGS and HGC-27 cells detected by Western blot analysis. The cell experiment was repeated three times. * *p* < 0.05 vs. sh-NC-treated HGC-27 cells, or Vector-treated AGS cells

Glutamine metabolism is involved in cancer metabolism, and the survival and proliferation of cancer cells are highly dependent on glutamine; as a key nitrogen and carbon donor, glutamine supplements the tricarboxylic acid cycle, and its metabolism can promote tumor progression [17, 18]. We subsequently aimed to verify the potential effects of circDYRK1A on glutamine metabolism in GC cells. As illustrated in Fig. 3A–C, Additional file 3: Fig. S2A–C, up-regulation of circDYRK1A inhibited the expression of glutamine, glutamic acid and α-KG in HGC-27 and AGS cells, while repression of circDYRK1A promoted the aforementioned expression in HGC-27 and AGS cells. In addition, there was a reduction in the protein levels of GLS and GDH in HGC-27 and AGS cells over-expressing circDYRK1A, while the opposite results were noted following depletion of circDYRK1A (Fig. 3D, Additional file 3: Fig. S2D). Overall, these findings indicated that circDYRK1A may restrict the glutamine metabolism of GC cells.

### Transcription factor RUNX3 promotes circDYRK1A expression and inhibits GC progression

**Fig. 4:**
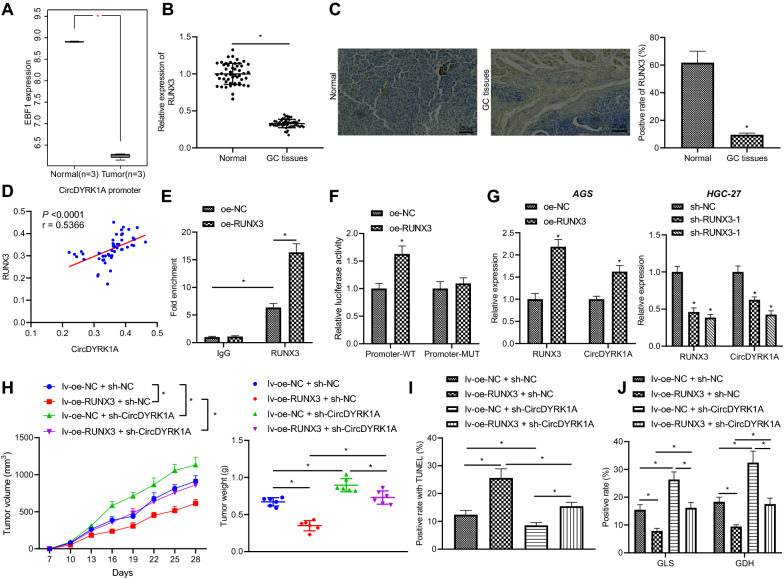
RUNX3 promotes the expression of circDYRK1A and inhibits the progression of GC. **A** The expression of RUNX3 in microarray dataset GSE49051. **B** The expression of RUNX3 in GC tissues and adjacent normal tissues detected by RT-qPCR (n = 50). **C** RUNX3 expression in GC tissues and adjacent normal tissues measured by immunohistochemistry. **D** Pearson correlation analysis of the correlation between RUNX3 expression and circDYRK1A expression in GC tissues. **E** The enrichment of RUNX3 in circDYRK1A promoter region detected by ChIP assay. **F** The effect of RUNX3 on circDYRK1A promoter activity. **G** Expression of RUNX3 and circDYRK1A in AGS and HGC-27 cells measured by dual luciferase reporter assay. **H** Tumor size (left panel), growth curve (middle panel) and weight (right panel) in each group of mice detected (n = 6). **I** The apoptosis of tumor cells detected by TUNEL staining. Cell nuclei were stained in blue by hematoxylin, and yellow–brown cells are positive cells. **J** The expression of GLS and GDH (n = 6) measured by immunohistochemistry. The cell experiment was repeated three times. * *p* < 0.05

In order to further explore the upstream regulation mechanism of circDYRK1A, five transcription factors (SPI1, RUNX3, PBX3, RUNX3 and STAT3) were obtained by predicting the transcription factors regulating circDYRK1A with the help of the TRCirc website. The expression of these five genes in GC was obtained by analyzing the GSE49051 microarray dataset (Additional file 1: Table S4). The RUNX3 gene exhibited the most profound down-regulation, and thus was chosen for further experimentation (Fig. 4A). Additionally, the results of RT-qPCR and immunohistochemistry (Fig. 4A–C) illustrated that RUNX3 expression was lower in GC tissues than that in adjacent normal tissues. In addition, Pearson correlation analysis demonstrated that RUNX3 expression was positively correlated with circDYRK1A expression in GC tissues, with the expression of RUNX3 in clinical tissues as the ordinate, and the expression of circDYRK1A as the abscissa (Fig. 4D).

Accordingly, we sought to investigate whether the transcription factor RUNX3 could bind to the promoter region of circDYRK1A. ChIP assay results (Fig. 4E) exhibited that RUNX3 was enriched in the promoter region of circDYRK1A, and the enrichment of RUNX3 in the promoter region was increased when RUNX3 was over-expressed. Meanwhile, the TRCirc website revealed that the binding site of RUNX3 in the circDYRK1A promoter region was (chr21:38,780,489–38,780,655). Thus, we constructed a luciferase reporter vector for the circDYRK1A promoter and a vector with mutation in the binding site, co-transfected luciferase reporter gene and RUNX3 expression vector into 293 T cells and measured luciferase activity 48 h after cell transfection using dual luciferase reporter assay. The obtained results demonstrated that over-expression of RUNX3 promoted the promoter activity of circDYRK1A (Fig. 4F). Furthermore, RT-qPCR results (Fig. 4G, Additional file 4: Fig. S3A, B) displayed that over-expression of RUNX3 in HGC-27 and AGS cells promoted the expression of circDYRK1A, while the expression of circDYRK1A was decreased following depletion of RUNX3. Overall, these findings suggested that RUNX3 can promote the expression of circDYRK1A in GC cells.

Thereafter, in order to illustrate that RUNX3 can regulate the progression of GC by promoting the transcription of circDYRK1A, HGC-27 cells were transduced with oe-RUNX3 and/or sh-circDYRK1A. Nude mice were subcutaneously injected with HGC-27 cells transduced with oe-RUNX3 and/or sh-circDYRK1A. Subsequent findings revealed that over-expression of RUNX3 restricted the growth of tumor in mice, while inhibition of circDYRK1A led to a promotion in tumor growth. On the other hand, both over-expression of RUNX3 and inhibition of circDYRK1A reversed the inhibitory effect of over-expression of RUNX3 alone (Fig. 4H).

TUNEL staining (Fig. 4I) illustrated that apoptosis was promoted in mice injected with HGC-27 cells transduced with oe-RUNX3, while reduced apoptosis was observed following depletion of circDYRK1A. The results of Immunohistochemistry (Fig. 4J) further displayed that the levels of GLS and GDH were reduced in mice injected with HGC-27 cells transduced with oe-RUNX3. Meanwhile, the opposite results were found in mice following depletion of circDYRK1A. Besides, there was a reduction in GLS and GDH levels in mice injected with HGC-27 cells transduced with oe-RUNX3 + sh-circDYRK1A than those with oe-RUNX3 alone. Therefore, these findings suggested that RUNX3 augmented the expression of circDYRK1A and impeded the progression of GC.

### CircDYRK1A acts as a sponge of miR-889-3p

**Fig. 5:**
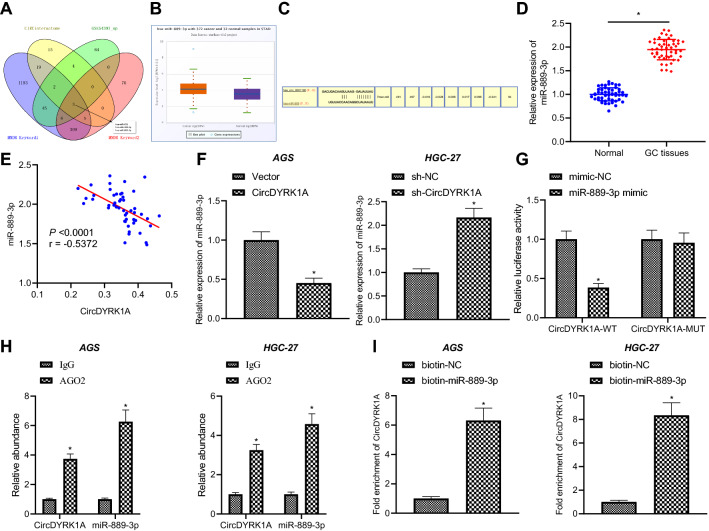
CircDYRK1A sponges miR-889-3p in GC cells. **A** Venn map of intersection of upregulated miRNA set among GC-related miRNA set through MNDR database, circDYRK1A targeted miRNA set predicted by Circinteractome database, and differential analysis of microarray dataset GSE54397. **B** hsa-miR-889-3p expression in STAD patients in starBase database. **C** Targeted binding sites of hsa_circRNA_001826 and hsa-miR-889-3p. **D** miR-889-3p expression in GC tissues and adjacent normal tissues determined by RT-qPCR (n = 50). **E** Pearson correlation analysis of the correlation between miR-889-3p expression and circDYRK1A expression in GC tissues. AGS cells were transduced with circDYRK1A overexpression vector and HGC-27 cells were transduced with sh-circDYRK1A. **F** miR-889-3p expression in AGS and HGC-27 cells determined by RT-qPCR. **G** The binding of miR-889-3p and circDYRK1A detected by dual luciferase reporter assay in 293 T cells. **H** The binding of miR-889-3p and circDYRK1A with AGO protein detected by RIP assay. **I** miR-889-3p pull down circDYRK1A detected by RNA pull down assay. The cell experiment was repeated three times. * *p* < 0.05 vs. adjacent normal tissues, 293 T cells treated with mimic-NC, sh-NC-treated HGC-27 cells, Vector-treated AGS cells, or AGS and HGC-27 cells treated with IgG, or biotin-NC

circRNAs are known to act as miRNA sponge molecules in the cytoplasm to isolate miRNAs and regulate subsequent gene expression [19, 20]. In order to explore the miRNAs that circDYRK1A may bind to, we obtained the intersection of the up-regulated miRNA set among GC-related miRNA set (keywords 1: stomach cancer, key words 2: gastric adenocarcinoma) using the MNDR database, circDYRK1A targeted miRNA set predicted by the Circinteractome database, and differential analysis of the GSE54397 microarray dataset. Subsequent findings revealed three miRNAs (namely, hsa-miR-421, hsa-miR-188-3p and hsa-miR-889-3p) (Fig. 5A). In order to further screen miRNAs, we explored the expression patterns of these three miRNAs in GC using the starBase database, which revealed that the expression difference of hsa-miR-889-3p was the most significant (Fig. 5B). Therefore, hsa-miR-889-3p was adopted as hsa_circRNA_001826 targeted binding miRNA of hsa_circRNA_001826.

In lieu of the aforementioned findings, we speculated that circDYRK1A could sponge miR-889-3p in GC (Fig. 5C). For verification, RT-qPCR was carried out to determine the expression patterns of miR-889-3p in adjacent normal tissues and GC tissues. Subsequent results illustrated that miR-889-3p was highly expressed in GC tissues relative to adjacent normal tissues (Fig. 5D). There was a negative correlation between circDYRK1A expression and miR-889-3p expression in GC tissues, with the expression of miR-899-3p in clinical tissues as the ordinate, and the expression of circDYRK1A as the abscissa (Fig. 5E). The results of RT-qPCR exhibited that the expression of miR-889-3p was decreased after over-expression of circDYRK1A in AGS and HGC-27 cells, while there was an increase in the expression of miR-889-3p following silencing of circDYRK1A (Fig. 5F, Additional file 5: Fig. S4A, B). Furthermore, luciferase activity of circDYRK1A-WT was inhibited by miR-889-3p mimic, which did not alter that of circDYRK1A-MUT (*p* > 0.05) (Fig. 5G), indicating that miR-889-3p could indeed interact with circDYRK1A. Meanwhile, existing evidence suggests that through RNA-induced silencing complex binds to MRes, and AGO2 serves as the key component. The findings of RIP assay showed that both circDYRK1A and miR-889-3p could bind to the AGO2 protein (Fig. 5H). Simultaneously, miR-889-3p could pull down circDYRK1A (Fig. 5I). Together, these findings indicated that circDYRK1A served as a miR-889-3p sponge.

### miR-889-3p reverses the inhibitory effect of circDYRK1A on GC cells

**Fig. 6:**
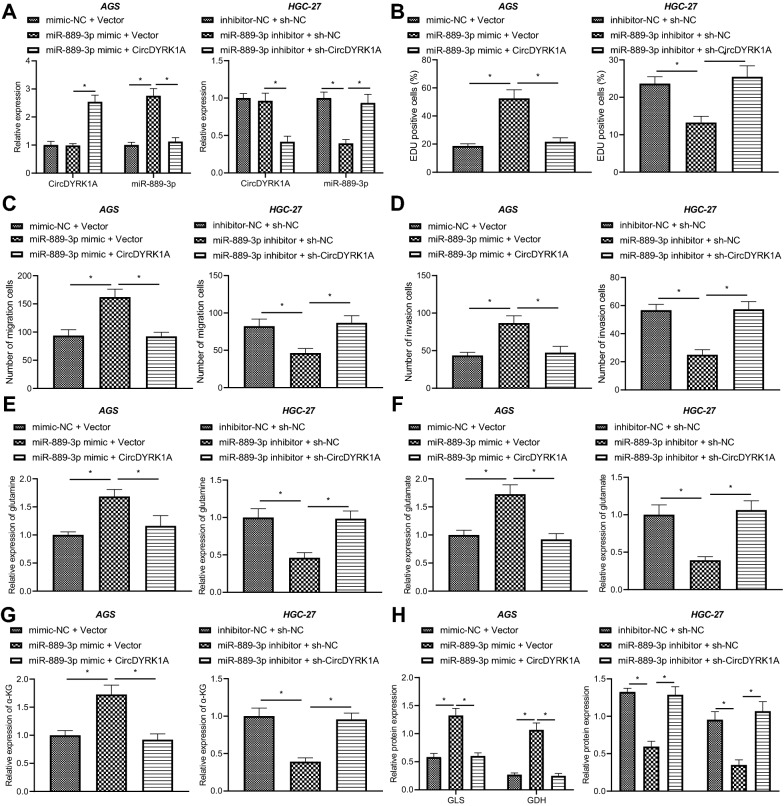
miR-889-3p reverses the inhibitory effect of circDYRK1A on malignant phenotypes of GC cells. AGS cells were transduced with miR-889-3p mimic and/or circDYRK1A, and HGC-27 cells were transduced with miR-889-3p inhibitor and/or sh-circDYRK1A. **A** Expression of circDYRK1A and miR-889-3p in AGS and HGC-27 cells determined by RT-qPCR. **B** Proliferation of AGS and HGC-27 cells detected by EdU assay. **C** Migration of AGS and HGC-27 cells detected by Transwell assay. **D** Invasion of AGS and HGC-27 cells detected by Transwell assay. **E** Expression of glutamine in AGS and HGC-27 cells measured using the kit. **F** Expression of glutamic acid in AGS and HGC-27 cells measured using the kit. **G** Expression of α-KG in AGS and HGC-27 cells measured using the kit. **H** Protein levels of GLS and GDH in AGS and HGC-27 cells measured by Western blot analysis. The cell experiment was repeated three times. * *p* < 0.05

Next, in an effort to investigate the effects of miR-889-3p, which was sponged by circDYRK1A, on the progression of GC, we transduced AGS and HGC-27 cells with miR-889-3p mimic and/or circDYRK1A, or miR-889-3p inhibitor and/or sh-circDYRK1A. Subsequent results of RT-qPCR (Fig. 6A, Additional file 6: Fig. S5A) illustrated that miR-889-3p expression was elevated, while the expression of circDYRK1A did not change significantly in HGC-27 and AGS cells transduced with miR-889-3p mimic. Relative to HGC-27 and AGS cells transduced with miR-889-3p mimic alone, miR-889-3p expression was decreased and circDYRK1A expression was increased in HGC-27 and AGS cells transduced with miR-889-3p mimic + circDYRK1A. Besides, there was a reduction in miR-889-3p expression while circDYRK1A expression showed no significant difference following miR-889-3p inhibition. Compared with miR-889-3p inhibitor alone, miR-889-3p expression was increased and circDYRK1A expression was diminished as a result of miR-889-3p inhibitor + sh-circDYRK1A treatment.

Furthermore, the results of EdU and Transwell assays (Fig. 6B–D, Additional file 6: Fig. S5B–D) illustrated that in HGC-27 and AGS cells, over-expression of miR-889-3p promoted the cell proliferative, migratory and invasion abilities, while over-expression of miR-889-3p and circDYRK1A impeded the malignant phenotypes of GC. In addition, repression of miR-889-3p inhibited the cell proliferative, migratory and invasion abilities, which were reversed by simultaneous inhibition of miR-889-3p and circDYRK1A. Moreover, we found that (Fig. 6E–G, Additional file 6: Fig. S5E–G) in HGC-27 and AGS cells, over-expression of miR-889-3p promoted the expression of glutamine, glutamic acid and α-KG, while up-regulation of miR-889-3p and circDYRK1A led to opposite results. Meanwhile, down-regulation of miR-889-3p inhibited the expression of glutamine, glutamic acid and α-KG, while these results were opposite following down-regulation of miR-889-3p and circDYRK1A. In addition, the results of Western blot analysis (Fig. 6H, Additional file 6: Fig. S5H) presented that the trends of GLS and GDH expression were the same as those of glutamine, glutamic acid and α-KG in AGS and HGC-27 cells, respectively. Specifically, over-expression of miR-889-3p promoted the expression of GLS and inhibited the expression of GDH, whereas simultaneous over-expression of miR-889-3p and circDYRK1A led to the opposite results. Altogether, these findings suggested that miR-889-3p could reverse the inhibitory effect of circDYRK1A on the proliferative, migratory and invasion abilities and glutamine metabolism of GC cells.

### CircDYRK1A up-regulates FBXO4 expression in GC cells by sponging miR-889-3p

**Fig. 7:**
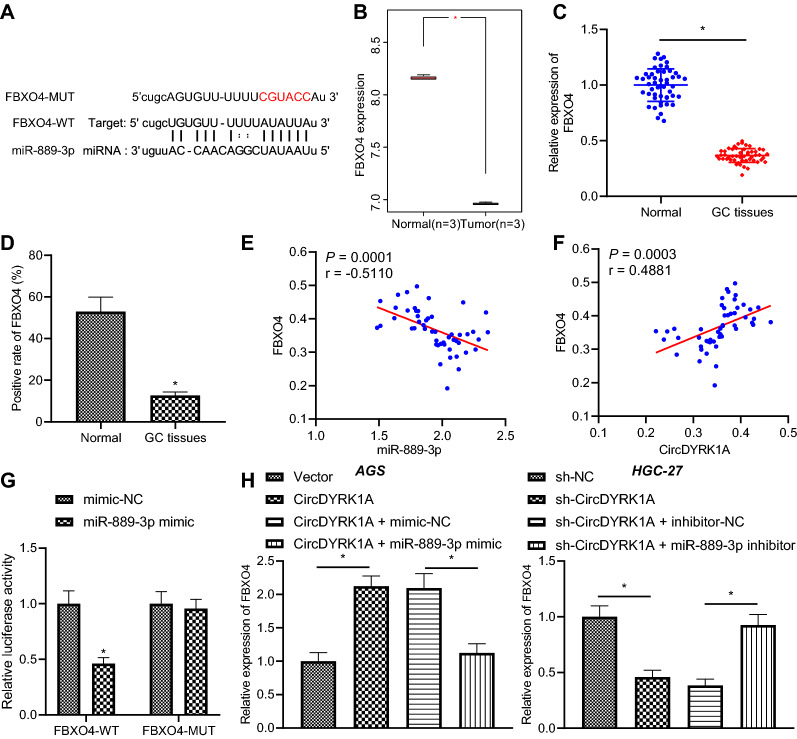
CircDYRK1A sponges miR-889-3p to promote FBXO4 expression. **A** The binding sites between miR-889-3p and FBXO4 predicted by starBase. **B** The expression of FBXO4 in GSE49051. **C** The expression of FBXO4 in GC tissues and adjacent normal tissues determined by RT-qPCR (n = 50). **D** The expression of FBXO4 in GC tissues and adjacent normal tissues determined by immunohistochemistry. **E** Pearson analysis of correlation between miR-889-3p and FBXO4. **F** Pearson analysis of correlation between circDYRK1A and FBXO4. **G** The correlation between miR-889-3p and FBXO4 detected by dual luciferase reporter assay. **H** The expression of FBXO4 in AGS and HGC-27 cells determined by RT-qPCR. The cell experiment was repeated three times. * *p* < 0.05

In order to further explore the mechanism of miR-889-3p in GC, we adopted the starBase database to predict the downstream target genes of miR-889-3p (Fig. 7A). It was found that FBXO4 expression was down-regulated in GC (Fig. 7B). Meanwhile, the results of RT-qPCR and immunohistochemistry illustrated lower expression of FBXO4 in GC tissues compared to that in adjacent normal tissues (Fig. 7C, D). Besides, the results of Pearson correlation analysis revealed that circDYRK1A expression was positively correlated with FBXO4 expression, while miR-889-3p expression exhibited an adverse relation with FBXO4 expression (Fig. 7E, F). Moreover, luciferase activity of FBXO4-WT was inhibited by miR-889-3p mimic, while there were no evident differences in the luciferase activity of FBXO4-MUT (*p* > 0.05) (Fig. 7G), indicating that miR-889-3p could specifically bind to the 3'UTR of FBXO4. Additionally, RT-qPCR results illustrated that over-expression of circDYRK1A in HGC-27 and AGS cells increased the FBXO4 expression, while over-expression of miR-889-3p and circDYRK1A reduced the expression of FBXO4. On the other hand, silencing of circDYRK1A in HGC-27 and AGS cells decreased the FBXO4 expression, whereas simultaneous reduction of miR-889-3p and circDYRK1A brought about an increase in FBXO4 expression (Fig. 7H, Additional file 7: Fig. S6A, B). Cumulatively, these findings indicated that circDYRK1A up-regulated FBXO4 expression by sponging miR-889-3p in GC cells.

### CircDYRK1A inhibits GC progression via miR-889-3p/FBXO4 axis

**Fig. 8:**
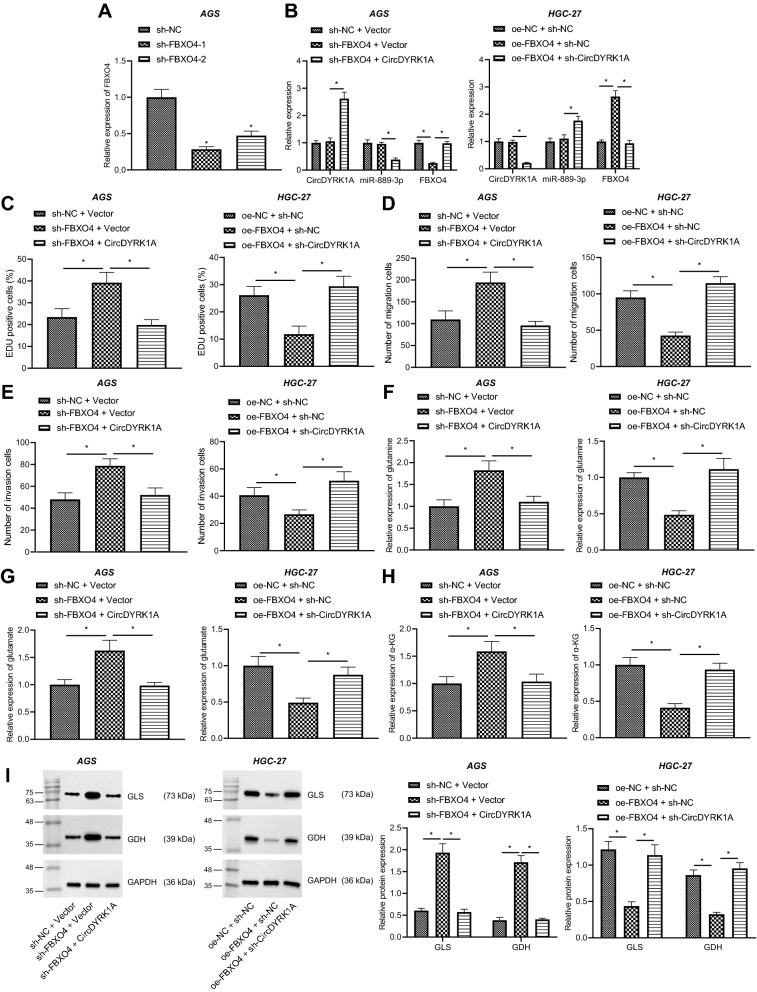
CircDYRK1A inhibits the malignant phenotypes and glutamine metabolism in GC cells by upregulating the expression of FBXO4 through miR-889-3p. AGS cells were transduced with sh-FBXO4-1 or sh-FBXO4-2. **A** Expression of FBXO4 in AGS cells determined by RT-qPCR. AGS cells were transduced with sh-FBXO4 and/or circDYRK1A, and HGC-27 cells were transduced with oe-FBXO4 and/or sh-circDYRK1A. **B** Expression of FBXO4, circDYRK1A and miR-889-3p in AGS cells and HGC-27 cells determined by RT-qPCR. **C** Proliferation of AGS cells and HGC-27 cells detected by EdU assay. **D** Migration of AGS and HGC-27 cells detected by Transwell assay. **E** Invasion of AGS and HGC-27 cells detected by Transwell assay. **F** Expression of glutamine in AGS and HGC-27 cells measured using the kit. **G** Expression of glutamic acid in AGS and HGC-27 cells measured using the kit. **H** Expression of α-KG in AGS and HGC-27 cells measured using the kit. **I** Protein levels of GLS and GDH in AGS and HGC-27 cells measured by Western blot analysis. The cell experiment was repeated three times. * *p* < 0.05

HGC-27 and AGS cells were further transduced with sh-FBXO4 and/or circDYRK1A, or oe-FBXO4 and/or sh-circDYRK1A in order to explore the effects of circDYRK1A/miR-889-3p/FBXO4 on GC progression. Subsequent results of RT-qPCR illustrated that FBXO4 expression was diminished in HGC-27 and AGS cells transduced with sh-FBXO4-1 and sh-FBXO4-2. Sh-FBXO4-1 exhibited the most profound silencing efficacy, and thus sh-FBXO4-1 was selected for further experiments (Fig. 8A, Additional file 8: Fig. S7A). Meanwhile, the results of RT-qPCR (Fig. 8B, Additional file 8: Fig. S7B) demonstrated that FBXO4 expression was diminished, while there were no significant changes in the expression of circDYRK1A and miR-889-3p following depletion of FBXO4. Relative to sh-FBXO4 alone, miR-889-3p expression was diminished, while the expression of circDYRK1A and FBXO4 was increased in the presence of sh-FBXO4 + circDYRK1A. Furthermore, FBXO4 expression was diminished, while expression of circDYRK1A and miR-889-3p showed no significant difference in HGC-27 and AGS cells transduced with oe-FBXO4. Compared with the HGC-27 and AGS cells transduced with oe-FBXO4 alone, there was an increase in miR-889-3p expression whereas expression of circDYRK1A and FBXO4 was decreased in HGC-27 and AGS cells transduced with oe-FBXO4 + sh-circDYRK1A. Besides, the results of EdU and Transwell assays (Fig. 8C–E, Additional file 8: Fig. S7C–E) revealed that in HGC-27 and AGS cells, down-regulation of FBXO4 promoted the cell proliferative, migratory and invasion abilities, while reduction of FBXO4 and over-expression of circDYRK1A reversed the malignant phenotypes. Besides, over-expression of FBXO4 inhibited the cell proliferative, migratory and invasion abilities, and the malignant phenotypes were reversed by up-regulation of FBXO4 and circDYRK1A knockdown. Moreover, we found that (Fig. 8F–H, Additional file 8: Fig. S7F–H) silencing of FBXO4 promoted the expression of glutamine, glutamic acid and α-KG in HGC-27 and AGS cells, whereas simultaneous silencing of FBXO4 and up-regulated circDYRK1A reversed these trends. In addition, restoring FBXO4 inhibited the expression of glutamine, glutamic acid and α-KG, while these trends were reversed after over-expression of FBXO4 in combination with reduction of circDYRK1A. Furthermore, the results of Western blot analysis (Fig. 8I, Additional file 8: Fig. S7I) illustrated that the trends of GLS and GDH levels were the same as that of glutamine, glutamic acid and α-KG in AGS and HGC-27 cells, respectively. Altogether, these findings confirmed that circDYRK1A up-regulated FBXO4 and suppressed the glutamine metabolism by sponging miR-889-3p, thereby inhibiting the progression of GC.

## Discussion

**Fig. 9:**
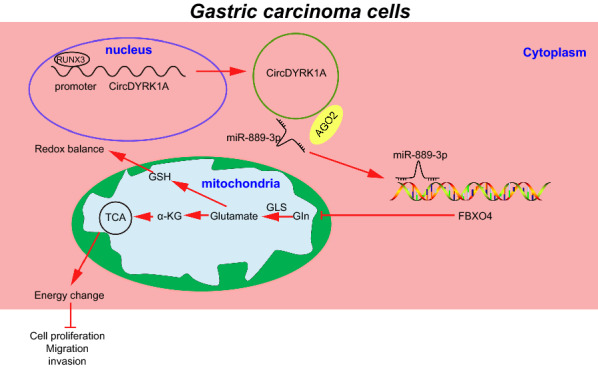
Graphical summary of mechanism of circDYRK1A in GC. RUNX3 enhances the expression of circDYRK1A, which in turn acts as a miR-889-3p sponge and upregulates FBXO4 expression, thus inhibiting glutamine metabolism and malignant phenotypes of GC cells to repress the progression of CG

Despite the downward trend in the incidence of GC, the malignancy remains a serious health burden owing to its high mortality [2]. Meanwhile, targeting glutamine metabolism in GC has been highlighted as an attractive strategy for the treatment of GC over the last few years [5]. However, there is a pressing need to elucidate the underlying molecular mechanisms and discovering potential biomarkers associated with glutamine metabolism in GC. Herein, findings obtained in our study uncovered the suppressive effect of RUNX3-mediated circDYRK1A on glutamine metabolism in GC via the miR-889-3p/FBXO4 axis (Fig. 9).

Firstly, initial findings in our study demonstrated that circDYRK1A was poorly expressed in GC tissues, and further associated with poor prognosis of GC patients. On the other hand, over-expression of circDYRK1A exerted a suppressive effect on glutamine metabolism, proliferative, migratory and invasion abilities of GC cells, thus arresting the progression of GC. Furthermore, there is plethora of evidence to suggest that circRNAs can exert varying effects on cell proliferation, survival, invasion and differentiation, in order to regulate tumorigenesis [21]. For instance, a prior study documented down-regulation of circ_100269 levels in GC tissues, whereas its over-expression led to inhibition of cell proliferation [22]. Similarly, circ-HuR (hsa_circ_0049027) was previously illustrated to be weakly expressed in GC tissues and cell lines, such that enforced expression of hsa_circ_0049027 suppressed the growth, invasion, and metastasis of GC cells in vitro and in vivo, thus retarding GC progression [23]. Further in line with our findings, another study has illustrated the ability of circ_0005075 to restrict cell growth and metastasis in GC, ultimately alleviating GC [9]. Meanwhile, glutamine, which can be transported into cells via transporters, is known to be converted to glutamate and further to α-KG by GDH, GLS and other enzymes for cancer cell growth and proliferation facilitation [17]. Moreover, existing evidence further indicates that glutaminolysis contributes to the survival and progression of tumor cells, and circRNAs can regulate cancer through glutamine metabolism [11]. Accordingly, it would be plausible to suggest that circDYRK1A could be adopted as a potential therapeutic target for the treatment of GC, underscoring the theoretical basis for the development of novel targets for GC.

Additionally, our findings further suggested that the RUNX3 gene could bind to the circDYRK1A promoter to promote circDYRK1A expression. RUNX3 is well-established as a putative tumor suppressor due to its ability to regulate the expression of a series of target genes, while RUNX3 down-regulation can induce the progression of GC [24]. In addition, RUNX3 was previously demonstrated to restrict GC progression and metastasis, and further highlighted as a suitable treatment target for GC [7]. Herein, our findings revealed that RUNX3-mediated circDYRK1A diminished the levels of glutamine, glutamic acid, and α-KG, and protein levels of GLS and GDH, and further restricted the cell proliferative, migratory and invasion abilities, collectively leading to suppression of GC progression. In lieu of the above-mentioned findings and evidences, targeting RUNX3-mediated circDYRK1A could aid the development of new therapeutic strategies for the prevention and treatment of GC.

Furthermore, we further uncovered that circDYRK1A could sponge miR-889-3p to down-regulate miR-889-3p expression and diminish the binding of miR-889-3p to FBXO4, thereby suppressing the glutamine metabolism and malignant phenotypes of GC cells. Interestingly, the hard-done work of our peers has shown that circRNAs can function as miRNA sponges in order to carry out regulation in a wide array of cancers [13]. For instance, a prior study indicated that circ_0001546 was capable of sponging miR-421 to restrict the proliferation and chemoresistance of GC cells [25]. Likewise, the investigation carried out by Cao et al*.* illustrated the ability of circLMO7 to act as a miR-30a-3p sponge to affect the glutamine metabolism, and also influence the malignant phenotypes of GC cells [13]. Furthermore, recent evidence has come to light revealing that miRNAs can promote GC cell malignant phenotypes, and thus facilitate GC development and progression, including miR-532 and miR-199a-3p [26, 27]. Herein, our study is the first-of-its-kind to reveal the promoting effect of miR-889-3p on the malignant phenotypes of GC cells, which can serve as a potential target for exploring novel therapeutic strategies against GC. Moreover, miRNAs are also capable of interacting with the 3’UTR of specific target genes, and consequently bring about a reduction of their expression [28]. In our study, the results of online biological prediction and luciferase reporter assay further identified that miR-889-3p bound to the 3’UTR of FBXO4 mRNA and inhibited its expression. The aforementioned findings represent the first evidence for post-transcriptional regulation of FBXO4 by miR-889-3p in GC cells and its potential importance in regulating GC progression. In addition, a recent study has revealed illustrated that reduction of FBXO4 facilitates carcinogen-induced papilloma development [29]. Additionally, aberrant expression of FBXO4 has been previously documented in various cancers with accumulation of cyclin D1 [30]. Besides, circRNAs are also established to serve as “miRNA sponges” to modulate gene expression [19]. Similarly, miRNAs are reported to regulate the expression of target genes to participate in the development of GC [15]. In light of the aforementioned evidence, we can conclusively suggest that circDYRK1A serves as a miR-889-3p sponge to attenuate the binding of miR-889-3p to FBXO4 and further up-regulates FBXO4 expression, thus affecting GC cell glutamine metabolism and malignant phenotypes.

## Conclusion

In summary, we demonstrated the tumor-inhibiting properties of RUNX3-mediated circDYRK1A in GC via regulation of miR-889-3p/FBXO4 at the molecular, cellular and animal levels. Our findings indicate that RUNX3-mediated circDYRK1A represses glutamine metabolism of GC cells and further restricts GC development by up-regulating miR-889-3p-dependent FBXO4. However, whether the therapeutic target is applicable in human beings requires further validation. In addition, it would be prudent to carry out large cohort studies to substantiate our findings, and, if so, targeting RUNX3-mediated circDYRK1A would offer a new therapeutic strategy against GC in the future.

## Supplementary Information

**Additional file 1:** **Table S1** Clinicopathological characteristics of patients with GC. **Table S2** Primer sequences for RT-qPCR. **Table S3** Expression of circRNAs in microarray datasets GSE89143 and GSE93541. **Table S4** Expression of five candidate genes in GC.

**Additional file 2: Fig. S1.** Overexpression of circDYRK1A inhibits malignant phenotypes of GC cells in vitro. HGC-27 cells were transduced with circDYRK1A overexpression vector and AGS cells were transduced with sh-circDYRK1A. A, Expression of circDYRK1A in AGS and HGC-27 cells determined by RT-qPCR. B, The proliferation of AGS and HGC-27 cells detected by EdU assay. C, The migration of AGS and HGC-27 cells detected by Transwell assay. D, The invasion of AGS and HGC-27 cells detected by Transwell assay. The cell experiment was repeated three times. * *p* < 0.05 *vs.* sh-NC-treated AGS cells or Vector-treated HGC-27 cells.

**Additional file 3: Fig. S2.** CircDYRK1A inhibits glutamine metabolism in GC cells. HGC-27 cells were transduced with circDYRK1A overexpression vector and AGS cells were transduced with sh-circDYRK1A. A, The expression of glutamine in AGS and HGC-27 cells detected using the kit. B, The expression of glutamic acid in AGS and HGC-27 cells detected using the kit. C, The expression of α-KG in AGS and HGC-27 cells detected using the kit. D, The protein levels of GLS and GDH in AGS and HGC-27 cells detected by Western blot analysis. The cell experiment was repeated three times. * *p* < 0.05 *vs.* sh-NC-treated AGS cells or Vector-treated HGC-27 cells.

**Additional file 4: Fig. S3.** Expression of RUNX3 and circDYRK1A in AGS and HGC-27 cells. A, Expression of RUNX3 and circDYRK1A in HGC-27 cells treated with oe-RUNX3 measured by RT-qPCR. B, Expression of RUNX3 and circDYRK1A in AGS cells treated with sh-RUNX3-1 or sh-RUNX3-2 measured by RT-qPCR. The cell experiment was repeated three times. * *p* < 0.05 *vs.* HGC-27 cells treated with oe-NC or AGS cells treated with sh-NC.

**Additional file 5: Fig. S4.** Expression of miR-889-3p in AGS and HGC-27 cells. A, Expression of miR-889-3p in HGC-27 cells treated with circDYRK1A measured by RT-qPCR. B, Expression of miR-889-3p in AGS cells treated with sh-circDYRK1A measured by RT-qPCR. The cell experiment was repeated three times. * *p* < 0.05 *vs.* HGC-27 cells treated with Vector or AGS cells treated with sh-NC.

**Additional file 6: Fig. S5.** miR-889-3p reverses the inhibitory effect of circDYRK1A on malignant phenotypes of GC cells. HGC-27 cells were transduced with miR-889-3p mimic and/or circDYRK1A, and AGS cells were transduced with miR-889-3p inhibitor and/or sh-circDYRK1A. A, Expression of circDYRK1A and miR-889-3p in AGS and HGC-27 cells determined by RT-qPCR. B, Proliferation of AGS and HGC-27 cells detected by EdU assay C, Migration of AGS and HGC-27 cells detected by Transwell assay. D, Invasion of AGS and HGC-27 cells detected by Transwell assay. E, Expression of glutamine in AGS and HGC-27 cells measured using the kit. F, Expression of glutamic acid in AGS and HGC-27 cells measured using the kit. G, Expression of α-KG in AGS and HGC-27 cells measured using the kit. H, Protein levels of GLS and GDH in AGS and HGC-27 cells measured by Western blot analysis. The cell experiment was repeated three times. * *p* < 0.05.

**Additional file 7: Fig. S6.** Expression of FBXO4 in AGS and HGC-27 cells. A, Expression of FBXO4 in HGC-27 cells treated with circDYRK1A or combined with miR-889-3p mimic determined by RT-qPCR. B, Expression of FBXO4 in AGS cells treated with sh-circDYRK1A or combined with miR-889-3p inhibitor determined by RT-qPCR. The cell experiment was repeated three times. * *p* < 0.05.

**Additional file 8: Fig. S7.** CircDYRK1A inhibits the malignant phenotypes and glutamine metabolism in GC cells by upregulating the expression of FBXO4 through miR-889-3p. HGC-27 cells were transduced with sh-FBXO4-1 or sh-FBXO4-2. A, Expression of FBXO4 in HGC-27 cells determined by RT-qPCR. HGC-27 cells were transduced with sh-FBXO4 and/or circDYRK1A, and AGS cells were transduced with oe-FBXO4 and/or sh-circDYRK1A. B, Expression of FBXO4, circDYRK1A and miR-889-3p in AGS cells and HGC-27 cells determined by RT-qPCR. C, Proliferation of AGS cells and HGC-27 cells detected by EdU assay. D, Migration of AGS and HGC-27 cells detected by Transwell assay. E, Invasion of AGS and HGC-27 cells detected by Transwell assay. F, Expression of glutamine in AGS and HGC-27 cells measured using the kit. G, Expression of glutamic acid in AGS and HGC-27 cells measured using the kit. H, Expression of α-KG in AGS and HGC-27 cells measured using the kit. I, Protein levels of GLS and GDH in AGS and HGC-27 cells measured by Western blot analysis. The cell experiment was repeated three times. * *p* < 0.05.

## Data Availability

All data generated or analyzed during this study are included in this article and its Additional files.

## Abbreviations

GC: Gastric cancer
ISH: In situ hybridization
NC: Negative control
RIP: RNA binding protein immunoprecipitation
FBA: Fetal bovine serum
DMEM: Dulbecco’s modified Eagle’s medium
RT-qPCR: Reverse transcription quantitative polymerase chain reaction
PVDF: Polyvinylidene difuoride
BSA: Bovine serum albumin
RIPA: Radio-immunoprecipitation assay
DAPI: 4', 6-Diamidino-2-phenylindole
FISH: Fluorescence in situ hybridization
ChIP: Chromatin immunoprecipitation
ANOVA: Analysis of variance
