# Supplementary material for: RUNX3-mediated circDYRK1A inhibits glutamine metabolism in gastric cancer by up-regulating microRNA-889-3p-dependent FBXO4
Source: J Transl Med. 2022 Mar 10;20:120. doi: 10.1186/s12967-022-03286-x (PMC8908664; doi:10.1186/s12967-022-03286-x)
Supplement: Supplementary file 1 — Additional file 1: Table S1 Clinicopathological characteristics of patients with GC. Table S2 Primer sequences for RT-qPCR. Table S3 Expression of circRNAs in microarray datasets GSE89143 and GSE93541. Table S4 Expression of five candidate genes in GC. [file 12967_2022_3286_MOESM1_ESM.docx]

**Table S1** Clinicopathological characteristics of patients with GC

| Characteristics | Case | CircDYRK1A expression | | *p* value |
| --- | --- | --- | --- | --- |
|  |  | Low | High |  |
| Age (year) |  |  |  | 0.3080 |
| < 60 | 16 | 6 | 10 |  |
| ≥ 60 | 34 | 18 | 16 |  |
| Gender |  |  |  | 0.4236 |
| Male | 37 | 19 | 18 |  |
| Female | 13 | 5 | 8 |  |
| Tumor size (cm) |  |  |  | 0.0005 |
| < 5 | 21 | 4 | 17 |  |
| ≥ 5 | 29 | 20 | 9 |  |
| TNM stage |  |  |  | 0.0062 |
| I + II | 18 | 4 | 14 |  |
| III + IV | 32 | 20 | 12 |  |
| Lymph node invasion |  |  |  | 0.0029 |
| Negative (N0) | 14 | 2 | 12 |  |
| Positive (N1-N3) | 36 | 22 | 14 |  |

**Table S2** Primer sequences for RT-qPCR

| Gene | Primer sequence |
| --- | --- |
| CircDYRK1A | Forward: 5′-AATCAGGCCTACCAGAATCGCCCAGTG-3′ |
|  | Reverse: 5′-AGAAAATTGGTATGTTGGATGTCCA-3′ |
| miR-889-3p | Forward: 5′-TTAATATCGGACAACCATTGT-3′ |
|  | Reverse: universal reverse primer provided by B532451 kit |
| U6 | Forward: U6 forward primer provided by B532451 kit |
|  | Reverse: universal reverse primer provided by B532451 kit |
| RUNX3 | Forward: 5’-ACCTCGGAACTGAACCCATT-3’ |
|  | Reverse: 5’-GGGAGGTAGGTATGGTGGAAG-3’ |
| FBXO4 | Forward: 5’-CCCTATCCACCCTCAGCTTT-3’ |
|  | Reverse: 5’-AGAACTCAGCATCCCCGAAA-3’ |
| GAPDH | Forward: 5’-GTGGACCTGACCTGCCGTCT-3’ |
|  | Reverse: 5’-GGAGGAGTGGGTGTCGCTGT-3’ |

**Table S3** Expression of circRNAs in microarray datasets GSE89143 and GSE93541

| circRNA | GSE89143 | | GSE93541 | |
| --- | --- | --- | --- | --- |
|  | logFC | adj. *p*. value | logFC | adj. *p.* value |
| hsa_circRNA_001826 | -2.42129 | 0.013942 | -7.18431 | 0.000344 |
| hsa_circRNA_101592 | -2.27912 | 0.011823 | -2.00737 | 0.011312 |
| hsa_circRNA_102417 | -2.07645 | 0.035415 | -2.22493 | 0.010711 |

**Table S4** Expression of five candidate genes in GC

| Gene | log2FC | *p*. value | adj. *p*. value |
| --- | --- | --- | --- |
| PBX3 | 2.200459 | 1.33E-10 | 7.94E-09 |
| RUNX3 | -4.45923 | 9.60E-10 | 2.52E-08 |
| EBF1 | -1.729 | 2.34E-08 | 2.22E-07 |
| STAT3 | -1.488 | 8.82E-06 | 2.78E-05 |
| SPI1 | -1.218 | 1.25E-06 | 5.22E-06 |
